# A Valuable Suggestion to Those Using Rubber Work in Their Practice

**Published:** 1862-02

**Authors:** E. Collins


### A VALUABLE SUGGESTION TO THOSE USING RUBBER WORK IN THEIR PRACTICE.

BY E. COLLINS.

After the work has been prepared in the ordinary way for flasking, place the piece upon a table, with palatal surface upward; encircle the piece with wax, cloth, or paper. This done, fill all interstices about the teeth with cotton to prevent the plaster from investing the crowns of the teeth when it is poured in upon the work: and thus may be avoided the slightest displacement in removing the model. This done, procure die and counter-die, with which swage a plate of gold or platina, (as no other metal will answer with impunity this purpose) which will cover the entire palatal surface; trimming it to fit properly the necks of the teeth. Next place the work upon the counter-die, punch holes about two lines in diamater around the alveolar border opposite to, and corresponding in number to the teeth. The instrument for this purpose should be tapering, having a square face, with a sharp edge, so that it will cut the plate, and leave an funnel shaped opening—also across the palatal border, and around the air chamber. If one should be used, swage the plate gently, and it is ready to be adjusted upon the work. When this is done, the openings should be filled with wax a little above the surface of the plate, which, when the work is warmed previous to packing, wlil be absorbed, leaving openings into which the gum is readily pressed, forming, when vulcanized, a perfect bond of union between the plate and the gum. The advantages which this improvement possesses over the ordinary style of executing rubber work, are as follows:

1st. To execute the work, one-third less gum over the palate is required; the strength imparted by the plate more than doubly compensates for the gum dispensed with; the gum need not be, over the vault of the palate, thicker than card paper.

2d. The plate possessing and retaining a polished surface, foreign matter can not so readily adhere to it as to the rubber. There is as much difference between the former and the latter, in this respect, as there is between a plow with a wooden mold-board and one with a mold-board of polished steel. One is a self-cleaner, and the other is a clean her yourself.

3d. The nerves of taste are not suffered to come in direct contact with the rubber, the surface of which is often so affected by the combined action of heat and moisture as to give rise to a most unpleasant astringent, bitter substance, of which the wearer often seriously complains. This, however, may be obviated wholly or in part, by letting the work go for a short time without cleaning, when it will be covered with an unctious gum which will exclude the moisture from it. The objections to the *Rubber Work*, as at present used, are such as to prevent its general adoption, but with the suggested improvement, is not only destined to be adopted into general use, but will be found to surpass gold work as far as continuous gum surpasses it and every other style of work n modern use.

